# Physical, Chemical, and Biological Properties of Chitosan-Coated Alginate Microparticles Loaded with Porcine Interleukin-1β: A Potential Protein Adjuvant Delivery System

**DOI:** 10.3390/ijms23179959

**Published:** 2022-09-01

**Authors:** Wan-Xuan Ho, Wen-Ting Chen, Chih-Hsuan Lien, Hsin-Yu Yang, Kuan-Hung Chen, Yu-Fan Wei, Meng-Han Wang, I-Ting Ko, Fan-Gang Tseng, Hsien-Sheng Yin

**Affiliations:** 1 Institute of Bioinformatics and Structural Biology, and College of Life Sciences, National Tsing Hua University, Hsinchu 30013, Taiwan; 2 Department of Engineering and System Science, National Tsing Hua University, Hsinchu 30013, Taiwan; 3 Nano Science and Technology Program, Taiwan International Graduate Program, Academia Sinica and National Tsing Hua University, Hsinchu 30013, Taiwan

**Keywords:** interleukin-1β, electrospray, zeta potential, alginate, chitosan, microparticles

## Abstract

We previously developed chicken interleukin-1β (IL-1β) mutants as single-dose adjuvants that induce protective immunity when co-administered with an avian vaccine. However, livestock such as pigs may require a vaccine adjuvant delivery system that provides long-lasting protection to reduce the need for successive booster doses. Therefore, we developed chitosan-coated alginate microparticles as a carrier for bovine serum albumin (BSA) or porcine IL-1β (pIL-1β) and assessed their physical, chemical, and biological properties. Electrospraying of the BSA-loaded alginate microparticles (BSA/ALG MPs) resulted in an encapsulation efficiency of 50%, and those MPs were then coated with chitosan (BSA/ALG/CHI MPs). Optical and scanning electron microscopy, zeta potential analysis, and Fourier transform infrared spectroscopy were used to characterize these MPs. The BSA encapsulation parameters were applied to ALG/CHI MPs loaded with pIL-1β, which were not cytotoxic to porcine fibroblasts but had enhanced bio-activity over unencapsulated pIL-1β. The chitosan layer of the BSA/ALG/CHI MPs prevented burst release and facilitated sustained release of pIL-1β for at least 28 days. In conclusion, BSA/ALG/CHI MPs prepared as a carrier for pIL-1β may be used as an adjuvant for the formulation of pig vaccines.

## 1. Introduction

Adjuvants have been used to enhance the immune response to vaccines for many decades [1]. Aluminum salts and oil emulsions are the most common adjuvants in veterinary vaccines [2]. However, aluminum salts are not effective for inducing cell-mediated immunity [3], and oil emulsions are prone to cause inflammation and ulcers at the injection site [4]. When included in an adjuvant preparation, cytokines can enhance primary and memory immune responses to certain types of infectious diseases [5]. The cytokine interleukin (IL)-1β is produced primarily by monocytes, macrophages, fibroblasts, and endothelial cells [6]. It not only activates its own gene but can also induce the cellular abundance of IL-6 and IL-8 [7]. Additionally, studies have shown that IL-1β is an effective adjuvant for humoral and cellular immune responses when co-administered with protein antigens in mice [8,9] and sheep [10,11]. We previously developed mutant chicken IL-1β as a single-dose vaccine adjuvant that accelerates and enhances mucosal immunity in combination with a vaccine targeting Newcastle disease virus. Moreover, a single dose of this IL-1β-containing vaccine induced protective immunity, with the added benefits of reduced animal stress and clinical cost [12]. The findings suggested that a mutant chicken IL-1β may be an effective adjuvant for vaccines used in the poultry industry. However, the vaccination of large livestock animals such as pigs and cattle may need booster doses to achieve maximum vaccine potency [13]. Moreover, vaccine costs should also be considered [14]. Therefore, a systematic approach is needed for development of adjuvants that may provide solutions to these issues for animals that are commonly treated at veterinary clinics.

Nanotechnology-derived vaccine delivery systems have received a substantial amount of attention during the last two decades as viable alternatives to conventional veterinary vaccines, although most are in the early stages of development [15,16]. Nevertheless, the use of microparticles (MPs) or nanoparticles as vaccine adjuvant delivery vehicles for veterinary medicine remains largely unexplored. Both MPs and nanoparticles can be manufactured from natural biodegradable polymers, which could help maintain the safety and quality of veterinary vaccine adjuvants [17,18]. For example, alginate is a natural polysaccharide that has been widely applied in the food and pharmaceutical industries because of its low toxicity, low price, and high biocompatibility [19]. Anionic alginate polysaccharides can form hydrogels in the presence of a divalent cation such as Ca^2+^ [20]. Alginate MPs have been successfully used for encapsulating vaccines for various animal species, including fish [21], mice [22], and cattle [23]. Nevertheless, the potential for prolonged drug release from sodium alginate particles is still limited owing to sodium alginate’s solubility, which leads to rapid particle dissolution in vivo [24]. However, a copolymer such as chitosan can be used to coat alginate MPs to reduce the initial “burst” release of an encapsulated drug or antigen [25]. Chitosan is a naturally occurring cationic polymer that is derived by deacetylating chitin in alkaline solution [26]. Because of its lack of toxicity as well as its biocompatibility and biodegradability, chitosan is highly suitable for pharmaceutical and biomedical applications [27]. It has been demonstrated that IL-1β abundance is robustly induced by priming mouse bone marrow-derived macrophages with 100 ng/mL lipopolysaccharide for 3 h, followed by 0.1 mg/mL chitosan for 6 h [28]. Additionally, high concentrations of sodium alginate (1 to 3 mg/mL) can increase IL-6 abundance in mouse macrophages [29]. Additionally, the cationic nature of chitosan can facilitate ionic interactions with the negatively charged carboxyl groups of alginate [30].

Polymers can provide substantial benefits to colloidal drug delivery systems because they help optimize drug loading and release [31,32]. Electrospraying is an efficient means of preparing nanoparticles via encapsulation for protein/drug delivery and other carrier-mediated systems [33]. This technique works by forcing a polymer solution through a syringe needle, which allows the solution to become charged via an electric field applied at the needle tip; hence, the solution is emitted as fine droplets. These charged droplets then fall towards an oppositely charged collector and generate MPs [34]. Electrospraying has a few advantages over other techniques for producing MP cargo carriers for various biomedical applications. Specifically, electrospraying produces particles with a narrow size distribution, i.e., from nanometers to microns [35], and the Coulombic repulsion inherent in this technique allows for self-dispersal of the highly charged electrosprayed droplets [36]. Although alginate and chitosan are widely used for the production of MPs and nanoparticles for drug delivery systems and have been extensively studied [37,38,39,40], alginate–chitosan MPs have yet to be fully characterized with respect to their use in veterinary vaccine adjuvants.

**Scheme 1:**
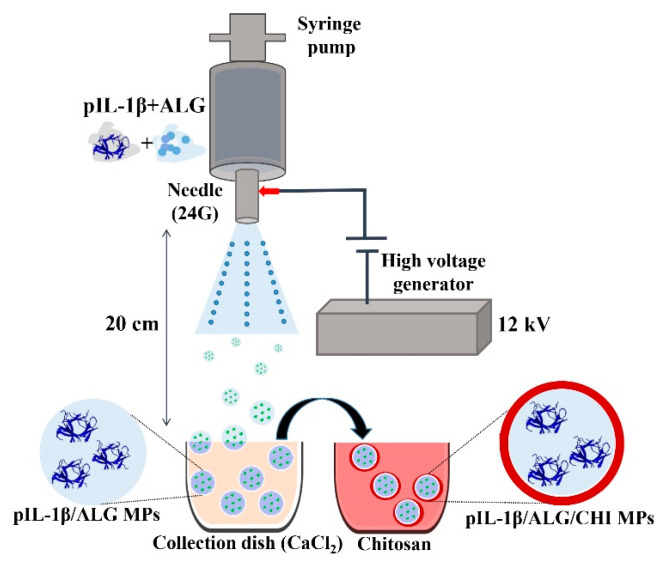
Schematic representation of the electrospraying setup and preparation of the chitosan-coated alginate MPs containing pIL-1β. A solution of sodium alginate containing recombinant pIL-1β protein was electrosprayed into CaCl_2_ solution and then pIL-1β/ALG MPs were formed. As a next step, the chitosan solution was combined with pIL-1β/ALG MPs in order to obtain pIL-1β/ALG/CHI MPs. In this study, a voltage of 12 kV, a flow rate of 0.1 mL/h, and a needle-to-collector distance of 20 cm were used. A description of the experimental details is provided in Section 3.2, Section 3.3, and Section 3.5.

Toward the goal of producing MPs to carry a cytokine adjuvant, here we used alginate MPs to encapsulate a model protein (bovine serum albumin, BSA) by cross-linking alginate with Ca^2+^ (in CaCl_2_ solution) using electrospray ionization, and encapsulation efficiency was evaluated. Alginate MPs were then further encapsulated in chitosan to form chitosan-coated, BSA-loaded alginate MPs (BSA/ALG/CHI MPs). Finally, chitosan-coated alginate MPs encapsulating recombinant porcine IL-1β (pIL-1β/ALG/CHI MPs) were fabricated using parameters derived from the BSA model (Scheme 1). The cytotoxicity, bioactivity, and release properties of these MP formulations were evaluated in vitro. By performing this study, we gained a deeper understanding of the properties of chitosan-coated alginate MPs as a potential delivery carrier, which could result in the sustained and prolonged release of pIL-1β and the enhancement of downstream IL-6 production. As veterinary vaccine adjuvant formulation strategies are crucial to achieving long-lasting immunity for large animals, such as pigs, it is imperative that effective vaccine adjuvant formulation strategies be devised. Veterinary practitioners may be able to achieve maximum vaccine potency and long-term immunity through the use of a microcarrier platform for protein adjuvant delivery.

## 2. Results and Discussion

### 2.1. Expression and Purification of IL-1β

**Figure 1:**
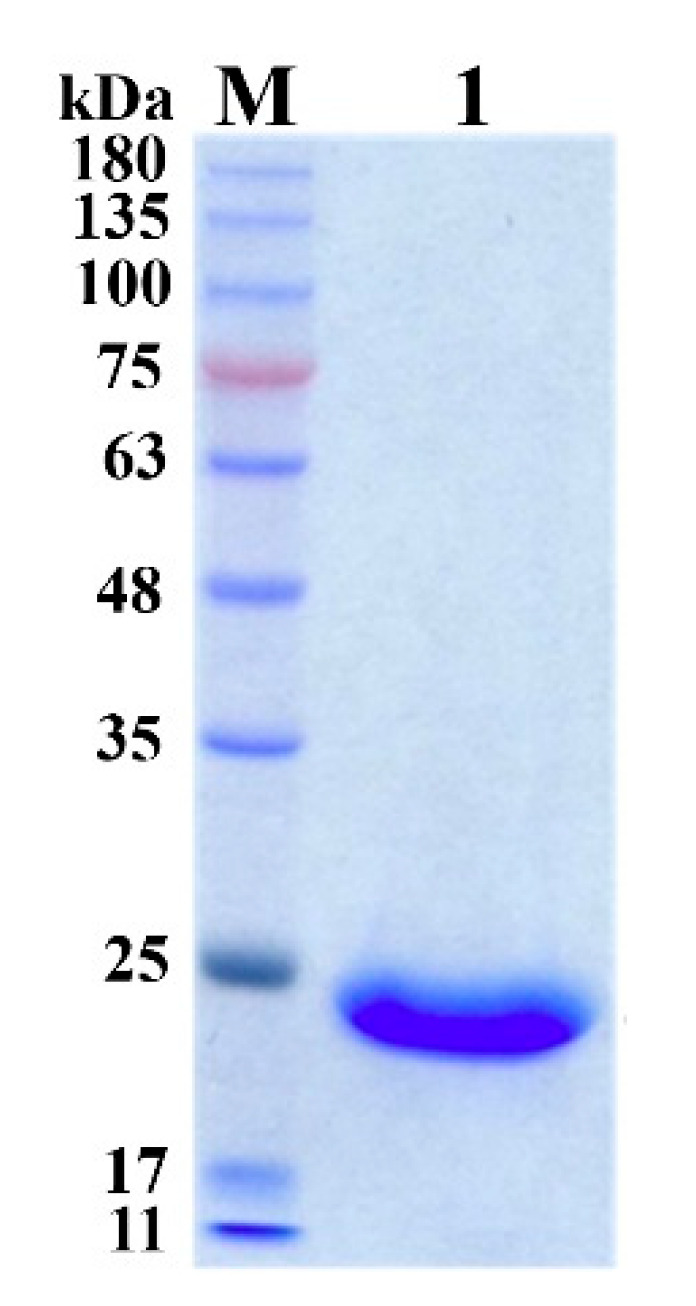
Purification of recombinant porcine interleukin 1-β (pIL-1β). The SDS–PAGE gel (15% (*w*/*v*) acrylamide) was stained with Coomassie blue. Lane 1, pIL-1β (10 µg); lane M, electrophoretic molecular mass markers.

The gene encoding pIL-1β was expressed in *E. coli* BL21 (DE3), and recombinant pIL-1β was purified to homogeneity through Co^2+^-affinity chromatography by using a His6 tag. The extracted pIL-1β was >99% pure as assessed by SDS–PAGE and Coomassie blue staining, and its molecular mass was approximately 20 kDa (Figure 1). This work presents the first report concerning the expression and purification of recombinant pIL-1β using a bacterial system. The purified pIL-1β was evaluated with regard to its potential as an MP-encapsulated bioadjuvant.

### 2.2. Efficiency of Encapsulating BSA in Alginate MPs

**Figure 2:**
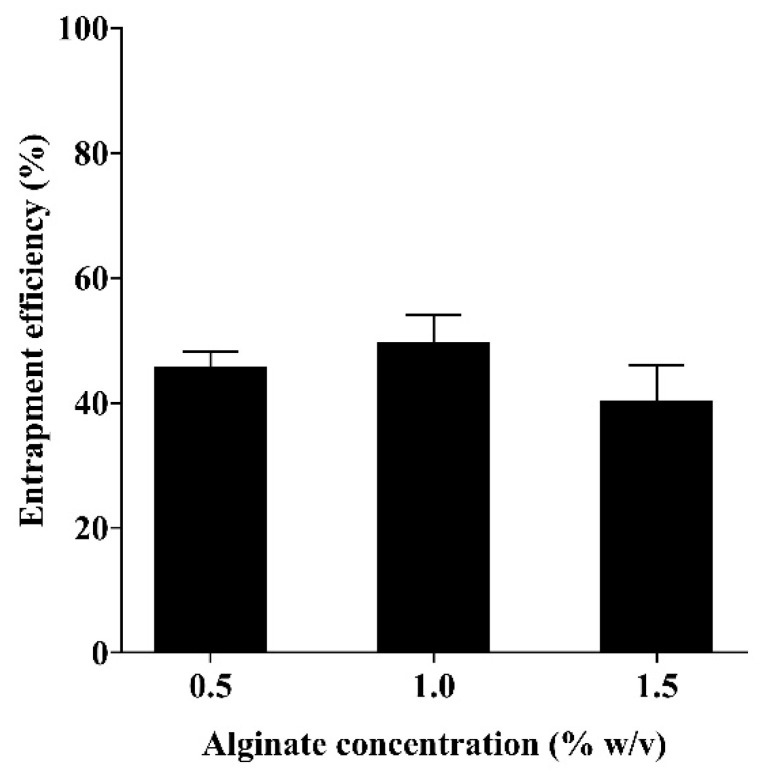
Encapsulation efficiency of bovine serum albumin (BSA)-loaded microparticles (MPs) at different concentrations of alginate.

Encapsulation efficiency was used to determine how much BSA could be encapsulated within alginate MPs at different MP concentrations. An alginate concentration of 1% (*w*/*v*) yielded the highest encapsulation efficiency for BSA, i.e., 49.7 ± 4.4%. Lower encapsulation efficiencies of 45.7 ± 2.6% and 40.2 ± 5.6% were estimated at 0.5% and 1.5% concentrations of alginate, respectively (Figure 2). Previous research had demonstrated that electrospraying BSA could produce monodispersed MPs with high encapsulation efficiency [41]. Indeed, another study found that electrospraying BSA plus alginate into a CaCl_2_ solution yielded an encapsulation efficiency ranging from 19.1% to 49.7% depending on the initial loading of BSA [42]. The latter percentage agreed with our encapsulation efficiency of 49.7% with 1% alginate; therefore, 1% alginate was utilized in subsequent experiments to optimize the inner encapsulation of alginate in MPs.

### 2.3. Microscopy of MPs

**Figure 3:**
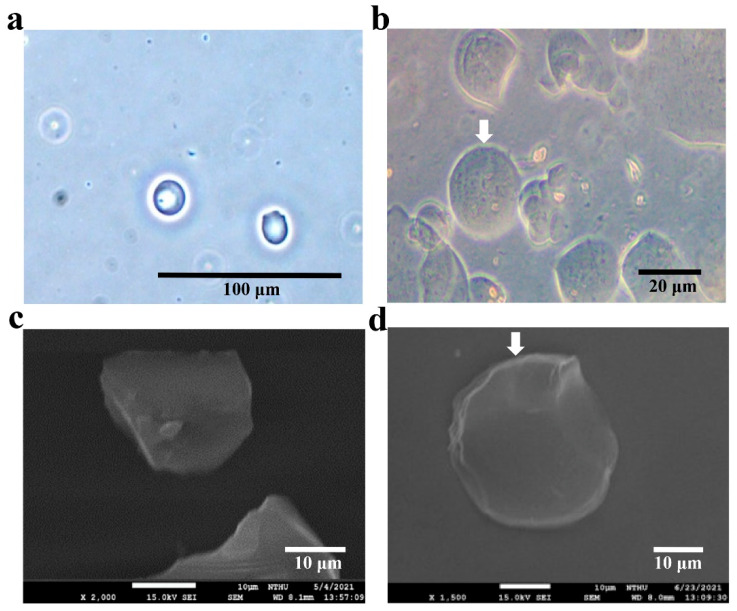
Optical microscopy (**a**,**b**) and scanning electron microscopy (**c**,**d**) of MPs. (**a**,**c**) Electrosprayed alginate MPs loaded with BSA. (**b**,**d**) BSA-loaded alginate MPs coated with chitosan. Scanning electron microscopy of (**c**) BSA/ALG/MPs with magnification 2000×; (**d**) BSA/ALG/CHI MPs with magnification 1500×; white scale bar: 10 μm. The white arrows indicate visible layers on the surface of alginate MPs.

To characterize the size distribution of our alginate-containing MPs, both BSA-loaded alginate MPs (BSA/ALG MPs and BSA/ALG/CHI MPs) were observed under optical and electron microscopes (Figure 3). Optical microscopy showed that the BSA/ALG MPs and BSA/ALG/CHI MPs were generally spherical with an approximate diameter of 20 μm (Figure 3a,b), suggesting that the MPs might be suitable for intramuscular delivery [43]. Scanning electron microscopy revealed that lyophilization of the BSA/ALG MPs resulted in irregularly shaped MPs (Figure 3c). However, the BSA/ALG/CHI MPs had relatively better sphericity and thus could withstand lyophilization better than the MPs that lacked the chitosan coating (Figure 3d). Indeed, research has demonstrated that sodium alginate is a colloid that swells in aqueous solution, and lyophilization destroys its spherical structure [44,45]. Previous work also demonstrated that sphericity improved by coating alginate-containing *Bifidobacterium longum* MPs [45]. Notably, layers of alginate were visible on the surface of our BSA/ALG/CHI MPs (Figure 3b,d), which may be a positive attribute of the chitosan coating [45,46].

### 2.4. Surface Charge of MPs

**Figure 4:**
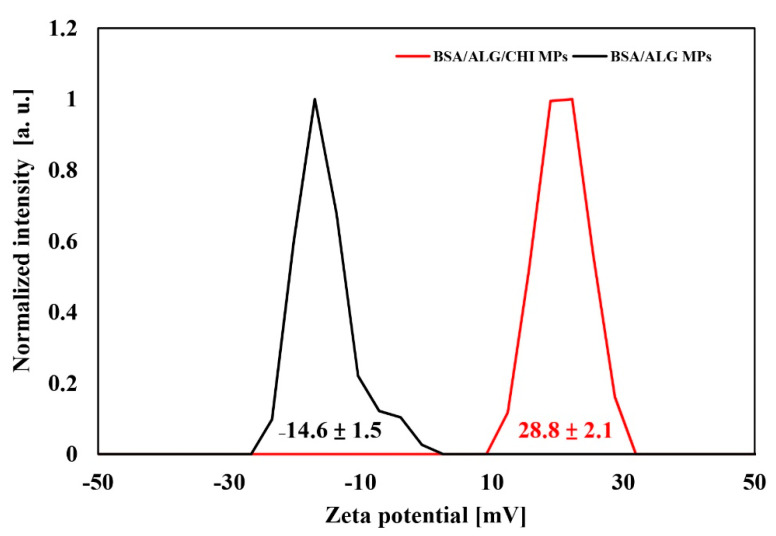
Zeta potential measurements for BSA-loaded alginate MPs (BSA/ALG MPs, black) and chitosan-coated BSA/ALG MPs (BSA/ALG/CHI MPs, red).

Zeta potential measurements revealed that BSA/ALG MPs were negatively charged (−14.6 ± 1.5 mV), but BSA/ALG/CHI MPs were positively charged (28.8 ± 2.1 mV) (Figure 4). This suggested that positively charged chitosan effectively interacted (electrostatically) with the negatively charged alginate surface. Zeta potential can be used to quantify the colloidal stability of MPs, and values greater than 30 mV or less than −30 mV indicate that the MPs are stable [47]. The BSA/ALG MPs had a zeta potential of −14.6 ± 1.5 mV, whereas the value for BSA/ALG/CHI MPs was 28.8 ± 2.1 mV (Figure 4), indicating that the MPs were colloidally stable in their final formulations. In addition, zeta potential also provides information regarding the surface charge of MPs and nanoparticles [48]. The switch of the zeta potential from −14.6 ± 1.5 mV (BSA/ALG MPs) to 28.8 ± 2.1 mV (BSA/ALG/CHI MPs) confirmed that a chitosan layer was present on the surface of the alginate MPs. The fact that this charge-switch phenomenon has previously been observed with alginate-coated chitosan MPs [49,50] confirmed that our MPs indeed had a chitosan outer shell.

### 2.5. FT-IR

**Figure 5:**
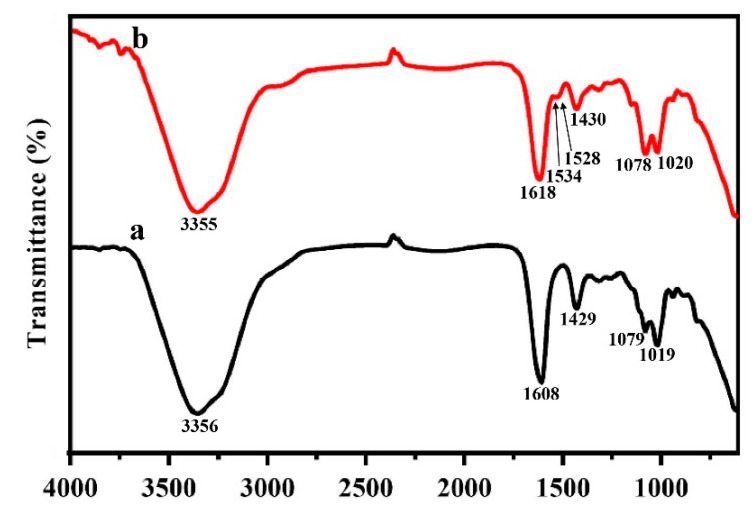
Fourier transform infrared spectra of BSA/ALG MPs (**a**) and BSA/ALG/CHI MPs (**b**).

FT-IR analysis was used to identify the functional groups present within the MPs. As shown in Figure 5, the spectrum of BSA/ALG MPs (Figure 5a) indicated that the peak observed at 3356 cm^−1^ corresponded to an O–H group [51], and this O–H stretching peak has also been reported for BSA encapsulated in alginate [52]. This result suggested that BSA was indeed encapsulated within alginate MPs. Peaks that we observed at 1608 cm^−1^ and 1429 cm^−1^ corresponded to –COOH asymmetric and –COOH symmetric stretching, respectively [53]. Peaks observed at 1079 cm^−1^ and 1019 cm^−1^ were characteristic of C–O and C–O–H bonds, respectively [54]. The spectrum of BSA/ALG/CHI MPs (Figure 5b) showed that the peak at 3355 cm^−1^ represented the stretching of N–H in chitosan [55,56]. Thus, the spectra of alginate and chitosan-coated alginate MPs showed a broad band at 3356–3355 cm^−1^, corresponding to O–H stretching that indicated intermolecular hydrogen bonding, and this broad band also overlapped with the N–H stretching in chitosan [56,57]. Complexation of alginate with chitosan resulted in spectral displacement of carboxylic groups, with peak shifts from 1608 and 1429 cm^−1^ to 1618 and 1430 cm^−1^, respectively. In the FT-IR spectrum of BSA/ALG/CHI MPs, the peak at 1528 cm^−1^ was indicative of N–H bending in amide groups in chitosan [58]. The corresponding peak in the range 1528–1534 cm^−1^ was attributed to C–N and C–N–H bending in chitosan [59,60]. In addition, the peak at 1078 cm^−1^ was assigned to C–O stretching, whereas the peak at 1020 cm^−1^ was attributed to C–O–C stretching, indicative of the structure of chitosan [61,62]. These results indicated that BSA was encapsulated within sodium alginate, and that the carboxyl groups of sodium alginate and amino groups of chitosan interacted electrostatically.

### 2.6. Cytotoxicity of MPs

**Figure 6:**
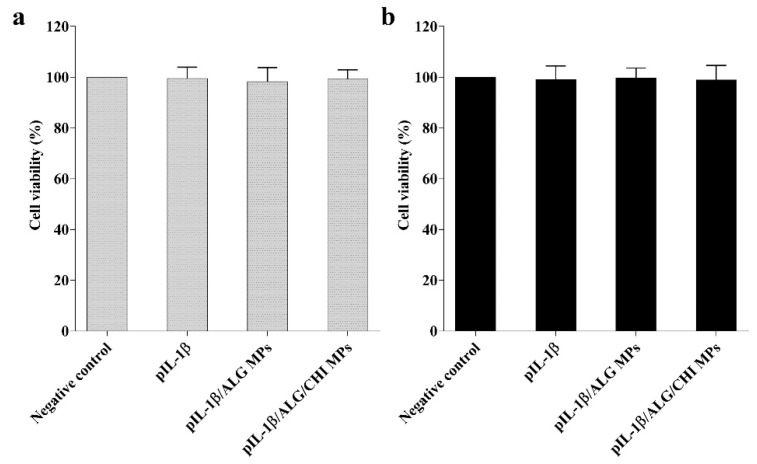
Cytotoxicity of pIL-1β, pIL-1β/ALG MPs, and pIL-1β/ALG/CHI MPs upon incubation with ST cells (**a**) or PK-15 cells (**b**). Negative controls included untreated ST cells and PK-15 cells.

The CCK-8 assay identifies viable cells by utilizing the ability of the mitochondrial dehydrogenases to oxidize the highly water soluble tetrazolium salt (WST)-8 to produce a colored formazan product [63]. CCK-8 assays were used to evaluate the cytotoxicity of pIL-1β, pIL-1β/ALG MPs, and pIL-1β/ALG/CHI MPs to ST cells (Figure 6a) and PK-15 cells (Figure 6b). Figure 6 presents data for the percentage of live cells for each treatment relative to the nontreated control cells. In comparison with the control ST cells, ST cells incubated with unencapsulated pIL-1 or with pIL-1β/ALG MPs or pIL-1β/ALG/CHI MPs had similar viabilities of 99.9 ± 4.5%, 98.2 ± 5.6%, and 99.3 ± 3.7%, respectively. Likewise, the treated PK-15 cells had similar relative viabilities of 99.1 ± 5.3%, 99.7 ± 4.1%, and 98.9 ± 5.7%, respectively. All these viability percentages were statistically indistinguishable (*p* > 0.05). These results indicated that neither pIL-1β nor any of the MP formulations prepared in this study were cytotoxic when examined in vitro. 

### 2.7. Effects of MP Formulations on IL-1β Signaling In Vitro

**Figure 7:**
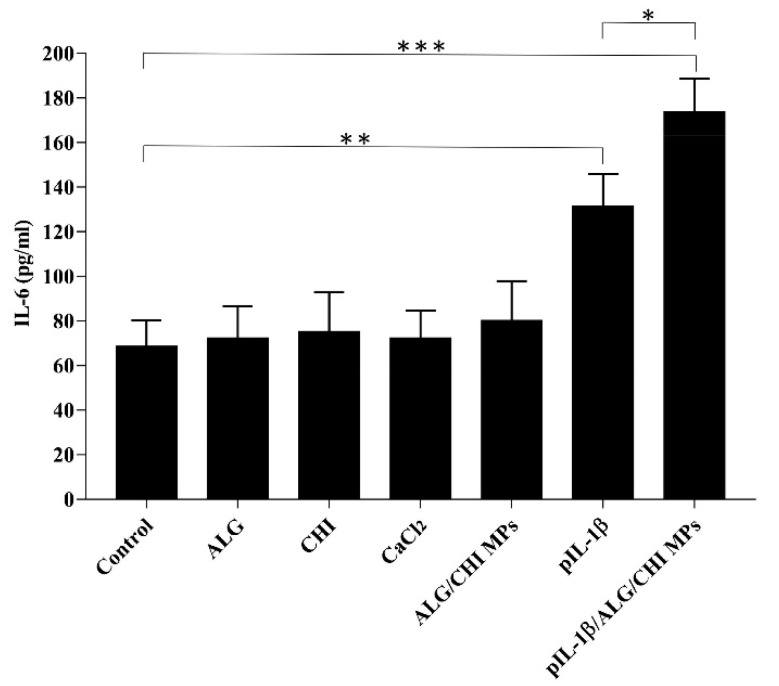
Effects of alginate, chitosan, CaCl_2_, ALG/CHI MPs, pIL-1β, and pIL-1β/ALG/CHI MPs on IL-6 abundance in lysates of the porcine fibroblast line ST. ST cells were cultured in Dulbecco’s Modified Eagle’s medium (Control) or 10 ng/mL of alginate, chitosan, CaCl_2_, ALG/CHI MPs, pIL-1β, or pIL-1β/ALG/CHI MPs for 8 h. Cells were lysed, and IL-6 abundance was measured using a porcine-specific ELISA. Data represent the mean ± standard deviation of triplicate determinations. * *p* < 0.05, pIL-1β vs. pIL-1β/CHI/ALG MPs; ** *p* < 0.01, pIL-1β vs. control; *** *p* < 0.001, pIL-1β/CHI/ALG MPs vs. control.

To evaluate the effects of MP formulations on IL-1β signaling in vitro, IL-6 abundance in lysates of the porcine fibroblast line ST was measured to assess pIL-1β-induced IL-6 production (Figure 7). At 10 ng/mL, unencapsulated pIL-1β significantly increased IL-6 abundance in the lysates (*p* < 0.01), whereas treatment with alginate, chitosan, CaCl_2_, or ALG/CHI MPs at the same molar equivalent had no effect on IL-6 abundance (*p* > 0.05). In contrast, the increase in IL-6 abundance induced by pIL-1β/ALG/CHI MPs was significantly higher than that observed in nontreated control cells (*p* < 0.001). pIL-1β/ALG/CHI MPs increased IL-6 abundance significantly relative to treatment with pIL-1β (*p* < 0.05). Mean IL-6 abundance induced by pIL-1β/ALG/CHI MPs relative to that induced by pIL-1β was 132%, indicating that the MP formulations may substantially enhance other porcine proinflammatory immune responses, at least as measured in vitro. Several factors, including polymer concentration, cell type, and stimulant may explain why alginate, chitosan, and ALG/CHI MPs were unable to significantly increase IL-6 abundance. One possibility is that pIL-1β/ALG/CHI MPs induced higher levels of IL-6 in fibroblasts than did unencapsulated pIL-1β because they protected interleukins from enzymatic degradation and sustained the stable release of pIL-1β [64,65]. Nevertheless, the results might also be explained by the fact that pIL-1β can also act as an immunostimulant, i.e., similar to lipopolysaccharide, which may have facilitated chitosan’s ability to enhance IL-1β abundance [28]. Thus, pIL-1β/ALG/CHI MPs increased IL-6 abundance in ST cells. A recent study revealed that alginate and chitosan are effective adjuvants for the hepatitis B vaccine in mouse models, which could reduce the dose of the this vaccine and thus be economically advantageous [66]. It may be necessary to increase the concentration of biopolymers to maximize the potential adjuvant-related effects of alginate-chitosan MPs, thereby reducing the dosage of the porcine vaccine. Thus, we suggest that porcine vaccines containing a triple-adjuvant formula with pIL-1β, alginate, and chitosan MPs might generate potent immunogenic responses in swine.

### 2.8. In Vitro Release from pIL-1β/ALG/CHI MPs

**Figure 8:**
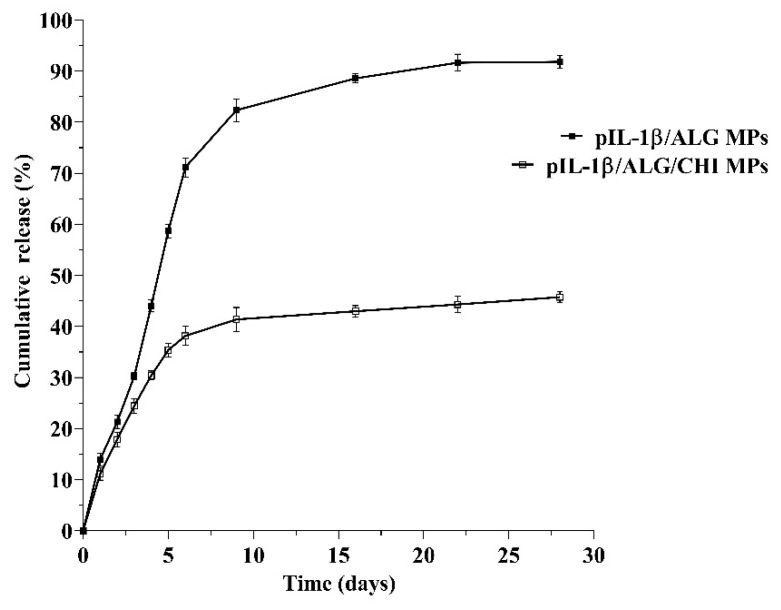
Cumulative release of pIL-1β from alginate MPs (■) and chitosan-coated alginate MPs (□) in PBS at pH 7.4. Data represent the mean ± standard deviation (*n* = 3).

One of the aims of this study was to slow down the release of pIL-1β by encapsulating it via two steps of microencapsulation. Therefore, we examined the rate of pIL-1β release from electrosprayed pIL-1β/ALG MPs and pIL-1β/ALG/CHI MPs. Figure 8 presents data for the cumulative release of pIL-1β from these two MP types at pH 7.4 over a period of 28 days. Notably, the initial “burst” release of pIL-1β from electrosprayed alginate MPs was not significant, totaling 13.9 ± 1.2% after 1 day, 30.3 ± 0.7% after 3 days, 71.2 ± 1.9% after 6 days, and = 91.8 ± 1.2% after 28 days. An important finding of this study was that the electrosprayed alginate MPs coated with chitosan exhibited a relatively slower, sustained release of pIL-1β, totaling 11.1 ± 1.2% after 1 day, 17.9 ± 1.4% after 3 days, and 35.4 ± 1.3% after 6 days. Moreover, the release rate was substantially slower during the subsequent 22 days, and indeed 45.8 ± 1.1% of the pIL-1β had been released at the end of the 28-day study. Therefore, the total amount of pIL-1β released after 28 days from chitosan-coated alginate MPs was significantly lower than that released from uncoated alginate particles. Owing to the fact that chitosan forms a film easily, chitosan-coated alginate particles reduce matrix swelling and prolong the drug release period in comparison with uncoated alginate particles [67]. Therefore, we propose that chitosan-coated alginate MPs could be administered to pigs intramuscularly as a long-lasting adjuvant delivery system. We therefore chose a pH of 7.4 and temperature of 37 °C, i.e., consistent with pig physiology, for evaluating the release of pIL-1β from uncoated and chitosan-coated alginate MPs. The release of pIL-1β from MPs may be influenced by factors such as formulation composition and the manufacturing process. A previous study demonstrated that electrostatic interactions between alginate and chitosan, specifically at 1% chitosan, resulted in significantly increased caffeine retention within particles [68]. The electrostatic interactions between two polymers may create a stronger network that could maintain its integrity in liquid medium. Our electrosprayed alginate MPs were also coated with 1% (*w*/*v*) chitosan, which facilitated electrostatic interactions that may meet the requirements for prolonging pIL-1β release. Additionally, a chitosan coating on alginate has been shown to facilitate more sustained release of a hydrophilic drug with a lesser initial burst release [25]. In our present study, the pIL-1β, which is hydrophilic, that had been encapsulated in chitosan-coated alginate MPs was released in a relatively slow, sustained manner, with a reduced initial burst of release. A previous study found that poly(lactic-co-glycolic acid) (PLGA) MPs loaded with drug had the desirable size range (20–60 μm) and that in vitro release was prolonged for at least 28 days [69]. Comparatively, intrasciatic nerve injection of PLGA-coupled MPs of 3.6 μm diameter resulted in MP dispersal within 2 weeks after injection [70]. These PLGA-coupled MPs had an approximate diameter of 20 μm, which may also have contributed to the prolongation of pIL-1β release. Although PLGA is also a suitable vehicle for prolonging drug release [71], the natural biopolymer composite in our MPs and the reduced initial burst release of pIL-1β (11.1 ± 1.2% release after 1 day) suggest that MPs may prove beneficial as adjuvant carriers in veterinary vaccines.

### 2.9. Stability of pIL-1β Released from MPs

**Figure 9:**
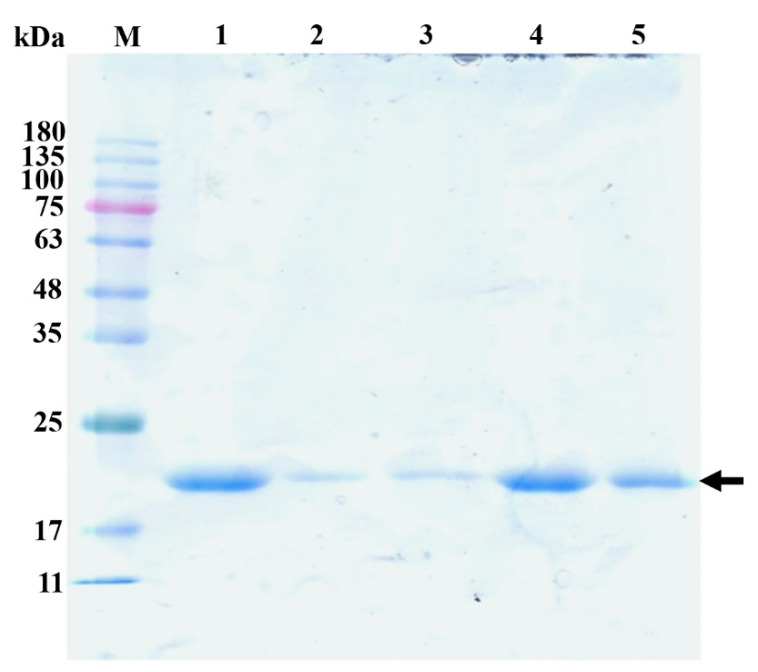
SDS–PAGE analysis of in vitro release assays. After 1 (lanes 2 and 3) and 6 (lanes 4 and 5) days, pIL-1β was released from alginate MPs (lanes 2 and 4) and chitosan-coated alginate MPs (lanes 3 and 5) compared to 5 µg of pure pIL-1β (lane 1). Lane M, electrophoretic molecular mass markers. The arrow indicates the pIL-1β.

In order to investigate whether any structural alternations were made to the pIL-1β after release from the ALG and ALG/CHI MPs, SDS–PAGE was performed (Figure 9). As shown in Figure 9, we found that a small amount of pIL-1β was present after 1 day of release from ALG (lane 2) or ALG/CHI (lane 3) MPs. A significant amount of pIL-1β was detected after 6 days of release from ALG MPs (lane 4) in comparison with ALG/CHI MPs (lane 5). Therefore, ALG/CHI MPs containing pIL-1β may release at a slower rate than ALG MPs containing pIL-1β. Additionally, a successful protein drug delivery system must be able to maintain the conformational integrity of the protein during drug delivery [72,73]. An SDS–PAGE analysis revealed no additional bands in the gel, such as protein aggregation or fragmentation. There was no lower or higher molecular weight band in the other lanes. Thus, the pIL-1β retains its primary structure during the release from the ALG and ALG/CHI MPs.

## 3. Materials and Methods

### 3.1. Materials

The sodium alginate (average molecular weight 155,000 Da, low viscosity, 15–20 cP for 1% in water at 25 °C), medium molecular weight chitosan (viscosity, 200–800 cP, 1 wt% in 1% acetic acid, molecular mass: 190,000–310,000 Da), BSA, CaCl_2_, and phosphate-buffered saline (PBS) were purchased from Sigma-Aldrich (St. Louis, MO, USA). *Escherichia coli* BL21 (DE3) strain was obtained from Yeastern Biotech Company (New Taipei City, Taiwan). Isopropyl β-d-1-thiogalactopyranoside was purchased from Protech (Taipei, Taiwan). Ampicillin, imidazole, NaCl, and tris(hydroxymethyl)aminomethane (Tris) were supplied by United States Biochemical (Cleveland, OH, USA).

### 3.2. Preparation of pIL-1β

The full-length cDNA for pIL-1β (GenBank accession number NM_214055) was synthesized by Genomics BioSci and Tech (Taipei, Taiwan). Subsequently, the gene encoding pIL-1β was cloned into pET-28a(+) (Novagen, Whitehouse Station, NJ, USA), with an upstream T7 promoter and histidine (His6) tag and expressed in *E. coli* BL21 (DE3). Bacteria were initially grown in Luria–Bertani medium containing 50 μg/mL ampicillin. After the OD_600_ had reached 0.6, isopropyl β-d-1-thiogalactopyranoside (0.4 mM final concentration) was added into the culture medium to induce protein expression for 4 h at 37 °C. To obtain soluble expressed proteins, whole cells were lysed by sonication in 25 mM Tris-HCl, 100 mM NaCl, and pH 7.4, and then centrifuged at 8000× *g* for 30 min at 4 °C. Recombinant His-tagged pIL-1β was purified by Co^2+^-affinity column chromatography (Clontech, CA, USA). After loading the *E. coli* extract, the column was initially washed with 100 mM imidazole, 25 mM Tris-HCl, 100 mM NaCl, pH 7.4, and His-tagged pIL-1β was eluted in the same solution but containing 300 mM imidazole. A Microcon YM-10 centrifugal filter unit with a 10,000 MW cut-off membrane (Millipore, Bedford, MA, USA) was then used to remove imidazole and to concentrate the protein. The purity of the purified pIL-1β was assessed by SDS-PAGE [74] and matrix-assisted laser desorption/ionization time-of-flight mass spectrometry (Autoflex III, Bruker Daltonics Inc., Billerica, MA, USA). Protein concentrations were determined using reagents of the Quick Start Bradford Protein Assay (Bio-Rad, Hercules, CA, USA) with BSA as the standard. PIL-1β (15 mg/mL) in 25 mM Tris-HCl, 100 mM NaCl, pH 7.4 was used as the stock solution.

### 3.3. Preparation of Alginate MPs

An alginate solution of 0.5%, 1.0%, or 1.5% *w*/*v*, and 10 mg/mL BSA [50] (or pIL-1β) was put into a 5 mL syringe with a metal needle (24 G) and connected to a high-voltage supply (CZE 1000R, Spellman High Voltage Electronics Corporation, Hauppauge, NY) set to 12 kV. The collector plate was 20 cm distant from the needle tip. A syringe pump (New Era Pump Systems Inc., NE-300, Hauppauge, NY, USA) controlled the solution feed rate at 0.1 mL/h. Droplets were dripped into a 0.3 M aqueous solution of CaCl_2_ for 30 min to allow the BSA/ALG or pIL-1β/ALG MPs to harden. The BSA/ALG or pIL-1β/ALG MPs were then washed with cold distilled water and pelleted down by centrifugation at 3000× *g* and 4 °C for 30 min. Afterward, the BSA/ALG or pIL-1β/ALG MPs suspensions were lyophilized for 48 h using a vacuum manifold freeze dryer (FDM-2, UNISS Technology Ltd., New Taipei City, Taiwan). The dried BSA/ALG or pIL-1β/ALG MPs were stored in glass vials at 4 °C.

### 3.4. Efficiency of Encapsulation

The Bradford method [75] was used to measure the encapsulation efficiency of MPs. The BSA-loaded ALG MPs were centrifuged at 10,000× *g* for 30 min at 4 °C to separate the MPs from the solution. The amount of free BSA present in the supernatant of collection solution was determined using Quick Start Bradford Protein Assay reagents with BSA as the standard. Encapsulation efficiency was calculated as (C_0_–C_1_/C_0_) × 100%, where C_0_ and C_1_ represent the total and free BSA concentrations, respectively. In all experiments, three replicates were performed.

### 3.5. Preparation of Chitosan-Coated Alginate MPs

Chitosan solution (1 wt% in 1% acetic acid) was added into the BSA/ALG or pIL-1β/ALG MPs suspensions and then sonicated (Model 3000; Misonix Inc, Farmingdale, NY, USA) for 10–30 s at 50 W [76], followed by gentle stirring with a magnetic stirrer for 2 h at 4 °C. Then the suspension was centrifuged at 1000× *g* for 10 min at 4 °C, and the supernatant was discarded. The BSA/ALG/CHI or pIL-1β/ALG/CHI MPs suspensions were lyophilized for 48 h using the FDM-2 freeze dryer. The dried BSA/ALG/CHI or pIL-1β/ALG/CHI MPs were stored at 4 °C.

### 3.6. Optical Microscopy

Microparticles were measured using an optical microscope to characterize their shapes and sizes. Using a sonicator at 3 W, the BSA/ALG and BSA/ALG/CHI MPs (1 mg/mL) were dispersed in distilled water for 60 s [77]. The dispersion was examined under a microscope (ZOOMKOP EZ-20I, New Taipei, Taiwan) by placing a drop on a glass slide. ImageJ software (National Institutes of Health, Bethesda, MD, USA) was used to binarize the images [78]. The mean diameter of each type of MPs was calculated as the average of 100 individual MPs.

### 3.7. Scanning Electron Microscopy

The size distributions of BSA/ALG and BSA/ALG/CHI MPs were examined under a scanning electron microscope (JEOL JSM-6380, Tokyo, Japan) at the National Tsing Hua University with an operating potential of 15 kV and width distance of 8.0 mm at 2000× and 1500× magnification, respectively. Prior to observation, samples were dried overnight on air at room temperature and then coated with a thin layer of gold [79].

### 3.8. Surface Charge

The surface zeta potential of BSA/ALG and BSA/ALG/CHI MPs was measured with a zeta-potential and particle-size analyzer (ELSZ-2000, Otsuka Electronics Ltd., Osaka, Japan) equipped with a 660 nm semiconductor laser operating a 15-degree detector angle. The measurements were conducted at a temperature of 25 °C. Each sample’s zeta potential was determined in triplicate based on its intensity distribution, and average values were calculated.

### 3.9. FT-IR Analysis

The BSA/ALG and BSA/ALG/CHI MPs were characterized by FT-IR spectroscopy (Nexus 870, Thermo Nicolet, WI, USA). A total of 2% (*w*/*w*) of sample was mixed with dry potassium bromide (KBr) (Sigma-Aldrich, St. Louis, MI, USA). The mixture was ground into a fine powder using an agate mortar before being compressed into KBr discs. KBr discs were scanned at 4 mm/s at a resolution of 2 cm over a wave number range of 640–4000 cm^−1^ [80].

### 3.10. Cytotoxicity Studies

A Cell-Counting Kit-8 (CCK-8) assay (Dojindo Molecular Technologies, Kumamoto, Japan) was used to evaluate in vitro cytotoxicity of alginate MPs with or without the chitosan coating in the swine testis cell line ST (ATCC CRL-1746) or porcine kidney cell line PK-15 (ATCC CCL-33), respectively. Cells were seeded into a 96-well plate (Nalge-Nunc International, Rochester, NY, USA) at 3 × 10^3^ cells per well and cultured in Dulbecco’s Modified Eagle’s medium at 37 °C with 5% CO_2_ for 18 h to facilitate adherence of cells. The medium was removed, and cells were treated for 24 h with 1 mg/mL MPs in culture medium: pIL-1β/ALG MPs, pIL-1β/ALG/CHI MPs, or pIL-1β; control cells were incubated in the absence of MPs. The medium was removed, and 100 μL of 10% CCK-8 was added with subsequent incubation for 2 h, after which the absorbance at 450 nm was measured for each well. Cell viability was expressed as a percentage relative to the nontreated control cells. All experiments were carried out in three independent assays with different batches of cells.

### 3.11. In Vitro Bioactivity of MP Formulations

The ST cells were seeded into a 6-well plate (Nalge-Nunc International) at 1.5 × 10^6^ cells per well and grown to 80–90% confluency in Dulbecco’s Modified Eagle’s medium. Cells were then washed with PBS and treated with CaCl_2_, alginate, chitosan, ALG/CHI MPs, pIL-1β, or pIL-1β/ALG/CHI MPs at a concentration of 10 ng/mL in culture medium. The cells plus reagents were incubated at 37 °C for 8 h. Control cells were incubated in medium without any added reagent. IL-6 abundance in cell lysates was determined using an IL-6-specific enzyme-linked immunosorbent assay (ELISA) kit (LifeSpan BioSciences, Seattle, WA, USA). The ELISA plate was read using the iMark microplate reader (Bio-Rad). All samples were evaluated in triplicate.

### 3.12. In Vitro Release of pIL-1β

The in vitro release of pIL-1β from pIL-1β/ALG MPs and pIL-1β/ALG/CHI MPs was evaluated as described previously [81] with some modifications. The MP suspensions were added to individual tubes containing PBS at 37 °C and placed in a shaker bath set to 50 rpm. Samples were taken over time and filtered through a low protein-binding filter (Millex 0.22 μm; Durapore polyvinylidene difluoride membrane; Merck Millipore, Darmstadt, Germany), followed by centrifugation at 8000× *g* for 20 min. Protein concentration in the supernatant was measured with Quick Start Bradford Protein Assay reagents. The percentage of pIL-1β released was determined based on the change in the concentration of pIL-1β measured over time.

### 3.13. SDS–PAGE of the Release pIL-1β

The primary structural integrity of released pIL-1β from ALG and ALG/CHI MPs after 1 day and 6 days in PBS (pH 7.4) was determined by SDS–PAGE and compared with pure pIL-1β and molecular weight markers. pIL-1β was present at 5 ug in both unencapsulated (pure) and encapsulated ALG and ALG/CHI MP samples. Aliquots (15 µL) of these dispersions were mixed with 4× sample buffer (0.25 mol/L Tris-HCl, pH 6.8, 80 g/L SDS, 200 mL/L glycerol, 100 g/L β-mercaptoethanol, and 1 g bromophenol blue) [82] denatured at 100 °C for 3 min. The gel (12% (*w*/*v*) acrylamide) was run under reducing conditions using Bio-Rad Mini-PROTEAN 3 Cell (Bio-Rad Laboratories) at a constant voltage mode of 120 V in a Tris/glycine/SDS buffer. The gel was stained with Coomassie blue staining solution and destained with 5% (*v*/*v*) acetic acid solution overnight.

### 3.14. Statistical Analysis

All experimental results were expressed as the mean value ± standard deviation. One-way analysis of variance was used to evaluate statistical differences among different groups using Prism software, version 7.00 (GraphPad Software, Inc. La Jolla, CA, USA). Differences between groups was evaluated statistically as *p* < 0.01 (**) or <0.001 (***).

## 4. Conclusions

To our knowledge, this is the first in vitro study to use a livestock cytokine encapsulated within a dual-layered chitosan-coated alginate MP, with subsequent characterization of its cytotoxicity, bioactivity, and release properties. Our findings suggest that the chitosan-coated alginate MPs could serve as a safe and effective delivery vehicle for long-lasting release of pIL-1β as well as for maintaining or improving its biological activity. MPs loaded with pIL-1β may possibly be employed as an adjuvant or immunostimulant for the formulation of vaccines for pigs.

## Data Availability

Not applicable.
